# Frequent interruptions of sedentary time modulates contraction- and insulin-stimulated glucose uptake pathways in muscle: Ancillary analysis from randomized clinical trials

**DOI:** 10.1038/srep32044

**Published:** 2016-08-24

**Authors:** Audrey Bergouignan, Celine Latouche, Sarah Heywood, Megan S. Grace, Medini Reddy-Luthmoodoo, Alaina K. Natoli, Neville Owen, David W. Dunstan, Bronwyn A. Kingwell

**Affiliations:** 1 Physical Activity Laboratory, Baker IDI Heart and Diabetes Institute, Melbourne, Victoria 3004, Australia; 2 Anschutz Health and Wellness Center, Division of Endocrinology, Metabolism and Diabetes, University of Colorado, Aurora, Colorado 80045, USA; 3 Metabolic and Vascular Physiology Laboratory, Baker IDI Heart and Diabetes Institute, Melbourne, Victoria 3004, Australia; 4 Department of Medicine, Monash University, Melbourne, Victoria 3800, Australia; 5 Behavioral Epidemiology Laboratory, Baker IDI Heart and Diabetes Institute, Melbourne, Victoria 3004, Australia; 6 Melbourne School of Population Health, The University of Melbourne, Melbourne, Victoria 3010, Australia; 7 School of Public Health, the University of Queensland, St Lucia, Queensland 4072, Australia; 8 School of Exercise and Nutrition Sciences, Deakin University, Burwood, Victoria 3125, Australia; 9 Department of Epidemiology and Preventive Medicine, Monash University, Melbourne, Victoria 3800, Australia; 10 Mary MacKillop Institute for Health Research, Australian Catholic University, Melbourne, Victoria 3000, Australia; 11 School of Sport Science, Exercise and Health, The University Of Western Australia, 35 Stirling Hwy, Crawley, Perth, Western Australia; 12 Department of Physiology, Monash University, Melbourne, Victoria 3800, Australia; 13 Department of Physiology, The University of Melbourne, Parkville, Victoria 3010, Australia

## Abstract

Epidemiological studies have observed associations between frequent interruptions of sitting time with physical activity bouts and beneficial metabolic outcomes, even in individuals who regularly exercise. Frequent interruptions to prolonged sitting reduce postprandial plasma glucose. Here we studied potential skeletal muscle mechanisms accounting for this improved control of glycemia in overweight adults under conditions of one day uninterrupted sitting and sitting interrupted with light-intensity or moderate-intensity walking every 20-min (n = 8); and, after three days of either uninterrupted sitting or light-intensity walking interruptions (n = 5). Contraction- and insulin-mediated glucose uptake signaling pathways as well as changes in oxidative phosphorylation proteins were examined. We showed that 1) both interventions reduce postprandial glucose concentration, 2) acute interruptions to sitting over one day stimulate the contraction-mediated glucose uptake pathway, 3) both acute interruptions to sitting with moderate-intensity activity over one day and light-intensity activity over three days induce a transition to modulation of the insulin-signaling pathway, in association with increased capacity for glucose transport. Only the moderate-intensity interruptions resulted in greater capacity for glycogen synthesis and likely for ATP production. These observations contribute to a mechanistic explanation of improved postprandial glucose metabolism with regular interruptions to sitting time, a promising preventive strategy for metabolic diseases.

Individuals at risk for developing type 2 diabetes (T2D) are more likely to spend a large proportion of the day in a state of hyperglycemia. Repeated hyperglycemic episodes can trigger an adverse biochemical cascade conducive to the development of diabetic and cardiovascular complications^1^,^2^. Regular moderate-to-vigorous exercise opposes multiple aspects of the pathophysiology of T2D and cardiovascular disease^3^,^4^,^5^,^6^. Interestingly, frequent interruptions to sitting time with bouts of physical activity are also associated with beneficial metabolic outcomes, even in individuals who regularly exercise^7^,^8^.

Based on these observations from population studies, we examined the acute effects of frequent interruptions to prolonged sitting with bouts of physical activity on postprandial plasma glucose and insulin concentrations. Using a cross-over design, we observed that overweight adults who interrupted their sitting with either light- or moderate-intensity bouts of walking every 20 min for 2 min over one day lowered postprandial glucose and insulin levels (−27% and −23%, respectively)^9^. Notably, no statistically-significant differences were observed between the light and moderate-intensity interventions. In a subsequent study, we examined the cumulative effects of the light-intensity intervention over three consecutive days. The decrease in plasma glucose and insulin induced by the frequent bouts of activity was sustained over three days of intervention^10^. Other groups have reported similar results^11^,^12^. However, the molecular mechanisms underlying the improvement in postprandial glycemic control have not been investigated.

Skeletal muscle is the main tissue responsible for plasma glucose disposal under postprandial conditions^13^. We have previously shown using a microarray approach that one-day of interrupting sitting is associated with changes in the expression of skeletal muscle genes involved in the regulation of carbohydrate metabolism^14^. However, the exact pathways that are modulated by briefly interrupting sitting time, including post-translational regulation, have not been delineated. Potential candidates include both the insulin- and contraction-mediated glucose uptake pathways. The insulin-mediated pathway involves PI3K (phosphoinositide 3-kinase) and subsequent phosphorylation of AKT^15^ and its downstream targets such as GSK3β and TBC1D4 (AKT substrate of 160 kDa, also called AS160)^16^. The latter is important in mediating translocation of the GLUT4 glucose transporter from intracellular compartments to the plasma membrane to promote cellular glucose uptake^17^. By inhibiting GSK3β (through phosphorylation), which inhibits glycogen synthesis^18^, insulin further regulates the metabolic fate of glucose between storage as glycogen and oxidation for ATP production. During muscle contraction, increases in the ADP/ATP and AMP/ATP ratios phosphorylate and activate AMPK (AMP-activated protein kinase)^19^, a heterotrimeric serine/threonine kinase whose activation switches off ATP-consuming pathways and switches on pathways that maintain ATP levels. Similar to AKT, AMPK phosphorylates TBC1D4, thereby promoting GLUT4 translocation to the membrane and glucose uptake into muscle^20^,^21^. AMPK activation also inhibits acetyl-CoA carboxylase (ACC) activity via an increase in ACC phosphorylation. The aim of this study was to investigate the potential molecular mechanisms accounting for the reduced post-prandial glucose concentrations observed with frequent interruptions to prolonged sitting. We examined the regulation of both insulin-mediated and contraction-dependent glucose disposal pathways in *vastus lateralis* muscle biopsies.

## Methods

### Study Overview

Data were derived from participants who were recruited for two laboratory-based cross-over design studies, which have been detailed elsewhere^9^,^10^. The “IDLE breaks” study examined the acute effect of light- and moderate-intensity breaks on postprandial insulin and glucose metabolism. Given similar effects of both light and the moderate-intensity interventions, the “ABLE breaks” study investigated the effect of light-intensity interruptions only over three days. These studies were carried out in accordance with the principles of the Declaration of Helsinki (2008), were approved by the Alfred Hospital Human Ethics Committee, and are registered as clinical trials with the Australian New Zealand Clinical Trials Registry (IDLE: ACTRN12609000656235 on the 04/08/2009 and ABLE: ACTRN12610000657022 on 11/08/2010). Written informed consent was obtained from each participant following an explanation of the experimental procedures and the risks involved. Consent was obtained for additional procedures for the subset of individuals who had biopsies.

### Participants

Recruitment and screening procedures have been detailed previously^9^,^10^. Briefly, in both studies eligible participants were aged between 45–75 years, with body mass index (BMI) >25 kg/m^2^, representing a population with a heightened risk for diabetes^9^ and were sedentary (self-reported sitting time >5 h/day). Exclusion criteria included clinically diagnosed diabetes, taking glucose- and/or lipid-lowering medication, smoking, and being physically active (>150 min/week moderate-intensity exercise for at least 3 months). Moderate-intensity walking speed was individually determined using the Borg Rate of Perceived Exertion [RPE] scale (rating between 12 and 14)^9^,^22^.

### Randomized control trials

The details of the methods and procedures used for randomization, assessment of the outcomes and estimation in these two randomized control trial studies have been fully explained previously^9^,^10^. Participants flowchart can be found in these two published manuscripts in which have been reported the main outcomes.

### Study Protocol

Study protocols for the IDLE^9^ and ABLE^10^ interventions were as follows, trial conditions being administered in random order:

#### 1-day protocol

Participants reported to the Alfred Center laboratory at 0700, having fasted overnight. An indwelling catheter was inserted into an antecubital vein for hourly blood collection to measure glucose and insulin concentrations. Following baseline blood draw, participants remained seated for 2 h to achieve a steady state before consuming a standardized test drink containing 75 g carbohydrate and 50 g fat. Participants were then guided through one of three trial condition protocols for the remaining 5 h:

- *Uninterrupted sitting*: participants remained seated throughout the experimental period, only rising from the chair to void.
- *Sitting plus light-intensity activity interruptions*: participants rose from the seated position every 20 min throughout the experimental period to complete a 2-min bout of light-intensity walking (3.2 km/h) on a motorized treadmill, providing a total of 28 min of light-intensity activity.
- *Sitting plus moderate-intensity activity interruptions*: identical to the sitting plus light-intensity interruptions condition, but participants completed 2-min bouts on the treadmill of moderate-intensity walking between 5.8 to 6.4 km/h every 20 min, providing a total of 28 min of moderate-intensity activity.

#### 3-day protocol

Participants arrived at 0800, having fasted overnight. On days 1 and 3, an indwelling catheter was inserted into an antecubital vein for the hourly collection of blood. The participants remained seated for the initial hour to achieve a steady-state before consuming a standardized test drink containing 75 g carbohydrate and 50 g fat, and completing one of two 6-h protocols:

- *Uninterrupted sitting*: participants remained seated throughout the experimental period on all three days, only rising from the chair to void. At the completion of the experimental protocol, participants were instructed to return home and limit movements to daily living activities only.
- *Sitting plus light-intensity activity interruptions*: participants rose from the seated position every 20 min throughout the experimental period to complete a 2-min bout of light-intensity walking (3.2 km/h) on a motorized treadmill, providing a total of 34 min/d of light-intensity activity.

Each condition was separated by a minimum of 6 days washout. Seven males and 1 female from the 19 participants in the IDLE study, and five males from the 19 participants in the ABLE study consented to give biopsy samples, which have been analyzed in the current investigation.

### Blood Samples

Plasma glucose and serum insulin were measured using hexokinase method and a chemiluminescent microparticle immunoassay, respectively (Architect ci16200 analyser, Abbott Diagnostics, North Ryde, NSW, Australia). The HOMA-IR (homeostasis model assessment of insulin resistance) index was calculated using fasting plasma glucose and insulin concentrations [glucose (mmol/l) × insulin (pmol/ml)]/135.

### Muscle biopsies

Biopsies were obtained from the *vastus lateralis* muscle using standard aseptic technique and local anesthesia approximately 20–30 minutes after final blood collection, which was approximately 40–50 minutes after the last activity bout. The biopsy samples were collected five and six hours after nutrient ingestion in the IDLE ‘1-day protocol’ and the ABLE ‘3-day protocol’ studies, respectively. A 7 mm skin incision was made, and the fascia opened. A side-cutting biopsy needle was passed through the incision to obtain roughly 100 mg of tissue under suction. All biopsies were snap frozen in liquid nitrogen and stored at −80 °C until further analysis.

### Western Blots

*Vastus lateralis* biopsy samples were homogenized in 8 μl of buffer per mg of tissue (20 mM HEPES, 2M EDTA, 50 mM NaF, 5 mM Na_4_P_2_O_7_, 1% NP40, 1 mM Na_3_VO_4_, 1 mM DTT, 0.05% SDS) containing a protease inhibitor cocktail (Roche Diagnostics, Meylan, France). The homogenates were centrifuged at 12,000 g for 10 min and the supernatant removed. Protein content was determined with the Pierce BCA Protein Assay (ThermoScientific, Rockford, IL, USA) and 30μg was loaded for each sample. Proteins were separated by gradient (4–20%) SDS-polyacrylamide gel electrophoresis (Novex, Carlsbad, CA, USA) and transferred to PVDF membranes (GE Healthcare, Rydalmere, NSW, Australia). Blots were blocked for 1 h at room temperature with 5% non-fat dry milk in TBS-T (20 mM Tris-HCl pH 8.0, 135 mM NaCl, 0.1% Tween-20). The membranes were subsequently incubated with anti-phosphoAKT (S473) (#4058, Cell Signaling Technology, Danvers, MA, USA), anti-totalAKT (#972, Cell Signaling Technology), anti-phosphoACC(S79) (#3661, Cell Signaling), anti-totalACC (#3662, Cell Signaling), anti-phosphoGSK3α/β(Ser21/9) (#9331, Cell Signaling Technology), anti-totalGSK3β (#9315, Cell Signaling Technology), or anti-oxidative phosphorylation complexes (OXPHOS) (#ab110413, Abcam, Waterloo, NSW, Australia) antibodies. Bound antibody was visualized using ECL Plus reagents (GE Healthcare) and signals were quantified using the Molecular Imager ChemiDoc and Quantity One 1-D analysis software (Bio-Rad Laboratories). Alpha-tubulin (#2144, Cell Signaling) was used as an internal control to further correct for protein for protein loading in all assays, but OXPHOS because the molecular weight of tubulin (52 kDa) was close to Complex V (55 kDa) and attempts at stripping failed. The full gels from the portions used in the figures are shown in the Supplemental Figures 1–7 and a summary of the Supplemental Figures can be found in the Supplemental Table 1.

### Statistical Analysis

Data are presented as mean ± SD, unless specified otherwise. Since data did not follow a normal distribution, the non-parametric Wilcoxon rank test was used to examine the effect of interruptions of prolonged sitting on post-transcriptional regulation of the metabolic pathways mediating skeletal muscle glucose uptake compared to the respective sedentary condition (normalised to 1). Statistical significance was defined at p < 0.05. Analyses were performed with SPSS software (v.22.0, IBM SPSS Statistics Inc., Chicago, IL).

## Results

### Participant characteristics

Participant characteristics are reported in Table 1. The eight participants recruited from the IDLE study had a mean age of 55 ± 6 yr and BMI of 31 ± 3 kg/m^2^. The five participants recruited from the ABLE study were on average 54 ± 6 yr and had a BMI of 29 ± 2 kg/m^2^. Participants were non-insulin resistant as shown by their HOMA-IR values within healthy human ranges.

### Postprandial plasma glucose and insulin concentration

**Figure 1:**
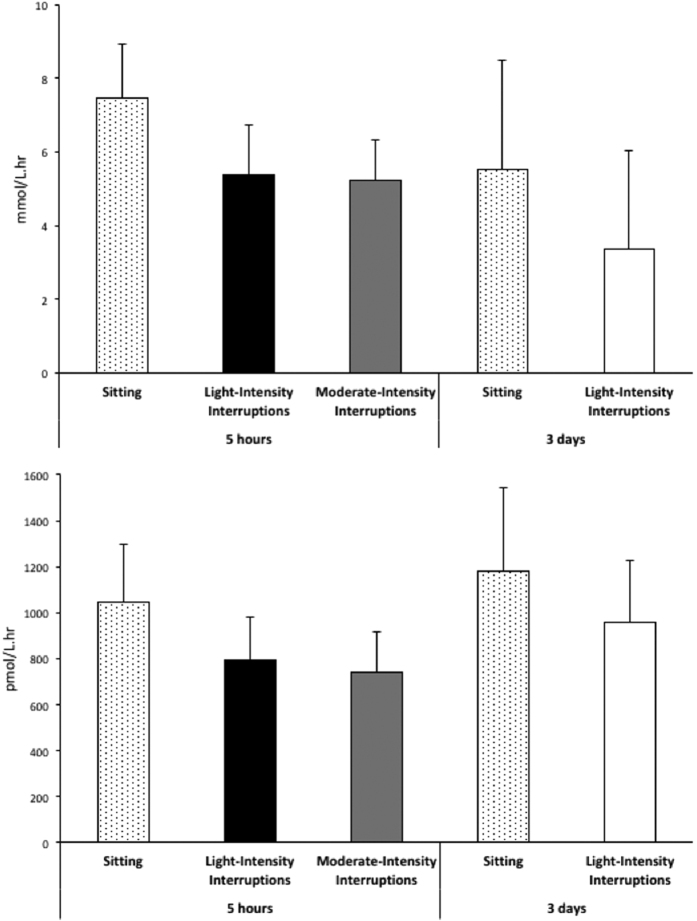
Effect of interrupting sitting time on postprandial plasma glucose and insulin incremental areas under the curve with 5 hr light and moderate-intensity bouts of activity (n = 8), or 3 days of light-intensity interruptions (n = 5). Data represent mean ± SEM.

As previously reported^9^,^10^, interrupting sitting with either light- or moderate-intensity walking decreased postprandial glucose (IDLE, light-intensity: −24%, p < 0.01; IDLE, moderate-intensity: −30%, p < 0.0001; ABLE: −31%, p = 0.001) and insulin (IDLE, both light- and moderate-intensity: −23%, p < 0.0001; ABLE: −15%, p = 0.001) incremental areas under the curve (iAUC) relative to uninterrupted sitting. In the subset of eight participants from the IDLE study reported in the current investigation, iAUC for glucose was reduced by 22% and 20% respectively with light- and moderate-intensity interruptions (Fig. 1). The iAUC for insulin was reduced by 25% and 24% after sitting interrupted with light-intensity and moderate-intensity walking relative to uninterrupted sitting, respectively. In the subset of five participants from the ABLE study reported in the current investigation, glucose and insulin iAUC were reduced by 25% and 19% respectively after three days of frequent interruptions of sitting with light-intensity walking compared to uninterrupted sitting.

### Contraction-mediated glucose uptake pathway

**Figure 2:**
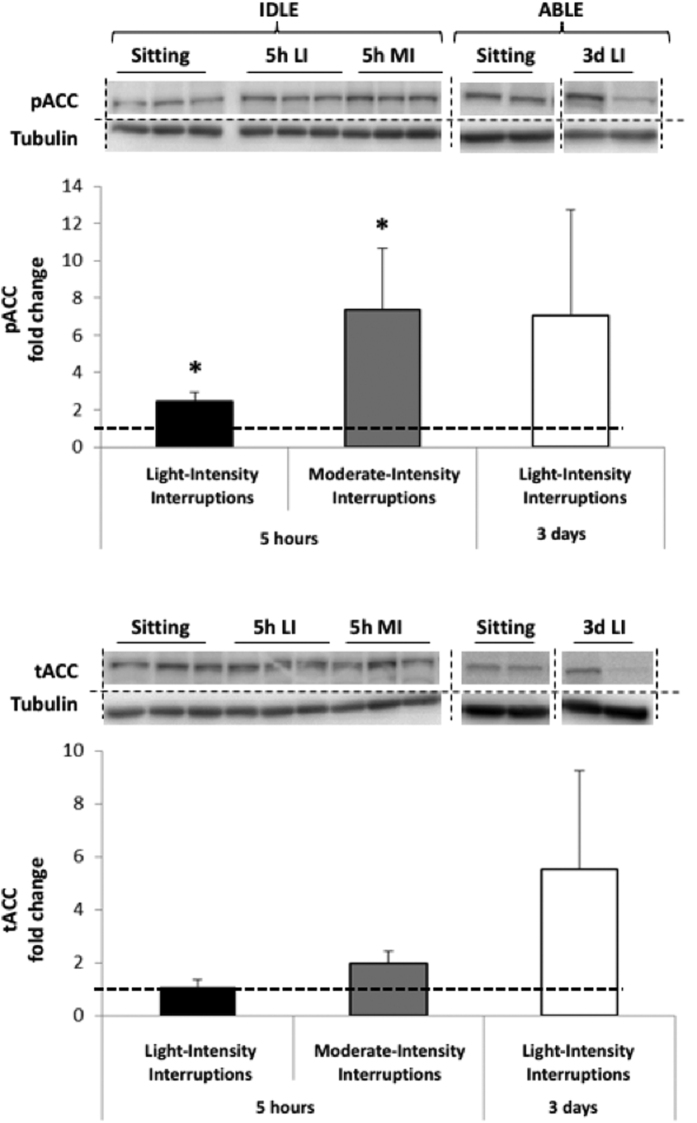
Changes in phosphorylated (Top) and total (Bottom) ACC protein expression in skeletal muscle following 5 hr light- (5h LI, n = 7) or moderate-intensity (5h MI, n = 7) interruptions, and 3 days of light-intensity interruptions (3d LI, n = 5) compared to uninterrupted sitting (value of 1). Data represent mean ± SEM. Representative blots are shown above the graphs. Dotted lines are showing where the images have been cropped; Full length blots/gels are presented in Supplemental Figures 1 (IDLE, ACC), 3 (IDLE, Tubulin), 4 (ABLE, ACC) and 5 (ABLE, Tubulin). Protein expression of ACC (280 kDa) and tubulin (52 kDa) have been measured on the same blot. All the blots have been run under exact same conditions. *P < 0.05 vs. sitting.

AMPK phosphorylation is typically transient and hard to capture, but results in more sustained phosphorylation of downstream ACC. We therefore used phosphorylation of the ACC protein as an index of activation of AMPK^23^,^24^,^25^ (Fig. 2), as previously reported. The 5 hr light- and moderate-intensity interruptions respectively increased pACC by 2.4 ± 1.1 (p = 0.040) and 7.3 ± 8.8 (p = 0.018) fold relative to uninterrupted sitting. There was no significant effect on tACC. Both pACC (7.0 ± 12.7 fold, p = 0.08) and tACC (5.5 ± 8.3 fold, p = 0.14) trended to increase after three days of light-intensity interruptions compared to the sedentary condition, but changes did not reach significance because of a large inter-individual variability. These changes however suggest an increased capacity for activation in response to three days of interruptions to prolonged sitting.

### Insulin-mediated glucose uptake pathway

**Figure 3:**
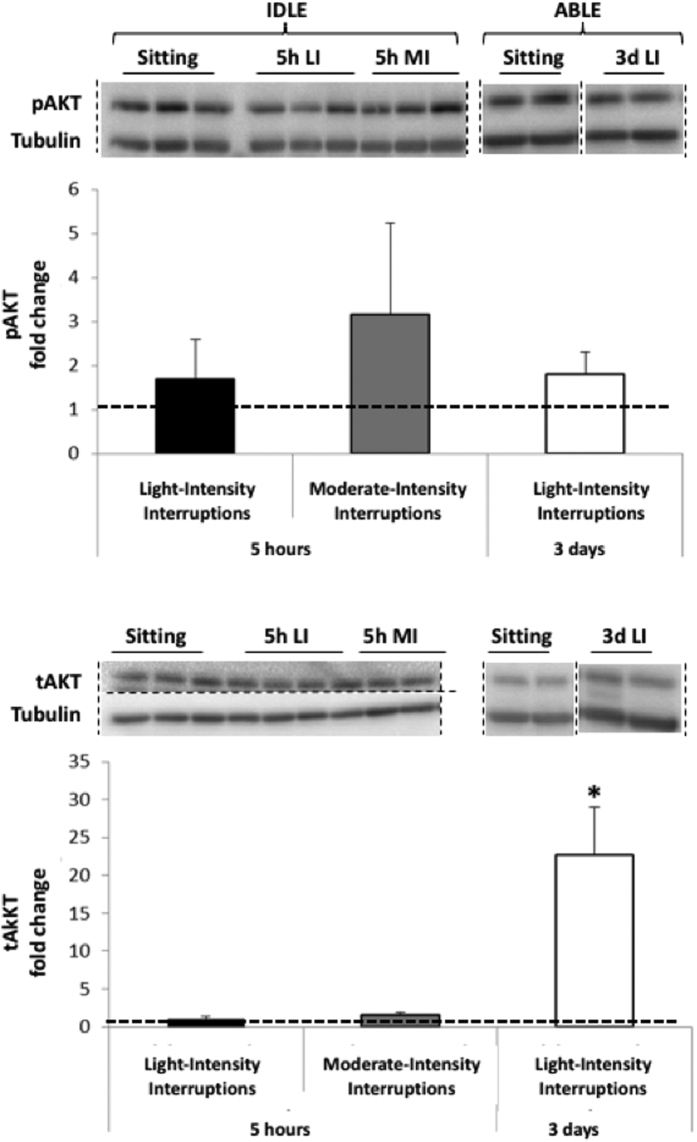
Changes in phosphorylated (Top) and total (Bottom) AKT protein expression in skeletal muscle following 5 hr light- (5h LI, n = 8) or moderate-intensity (5h MI, n = 8) interruptions, or 3 days of light-intensity interruptions (3d LI, n = 5) compared to uninterrupted sitting (value of 1). Data represent mean ± SEM. Representative blots are shown above the graphs. Dotted lines are showing where the images have been cropped; Full length blots/gels are presented in Supplemental Figures 1 (IDLE, AKT), 3 (IDLE, Tubulin), 4 (ABLE, AKT) and 5 (ABLE, Tubulin). Protein expression of AKT (60 kDa) and tubulin (52 kDa) have been measured on the same blot. All the blots have been run under exact same conditions. *P < 0.05 vs. sitting.

**Figure 4:**
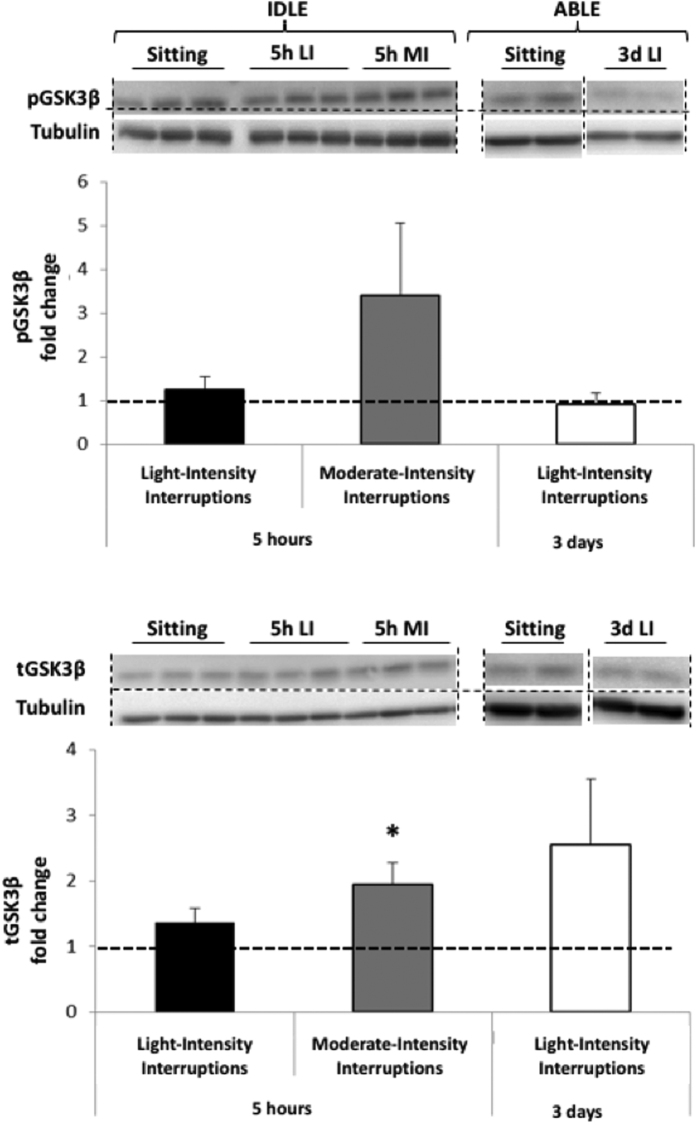
Changes in phosphorylated GSK3β (Top) and total (Bottom) GSK3β protein expression in skeletal muscle following 5 hr light- (5h LI, n = 7) or moderate-intensity (5h MI, n = 7) interruptions, or 3 days of light-intensity interruptions (3d LI, n = 5) compared to uninterrupted sitting (value of 1). Data represent mean ± SEM. Representative blots are shown above the graphs. Dotted lines are showing where the images have been cropped; Full length blots/gels are presented in Supplemental Figures 2 (IDLE, GSK3β), 3 (IDLE, Tubulin) and 5 (ABLE, GSK3β and Tubulin). Protein expression of GSK3β (46 kDa) and tubulin (52 kDa) have been measured on the same blot. All the blots have been run under exact same conditions. *P < 0.05 vs. sitting.

The insulin-mediated glucose uptake pathway was examined by assessing changes in phosphorylated and total amounts of AKT protein (Fig. 3). Neither the 5 hr light- nor moderate-intensity interruptions modified pAKT, tAKT and the downstream target, pGSK3αβ (Fig. 4). TotalGSK3β was increased in response to moderate-intensity interruptions (p = 0.036) but not to light-intensity interruptions. Three days of light-intensity interruptions did not change pGSK3αβ or tGSK3β, but increased tAKT 23 fold (p = 0.043) and tended to elevate pAKT (p = 0.14).

### Glucose transport signaling

**Figure 5:**
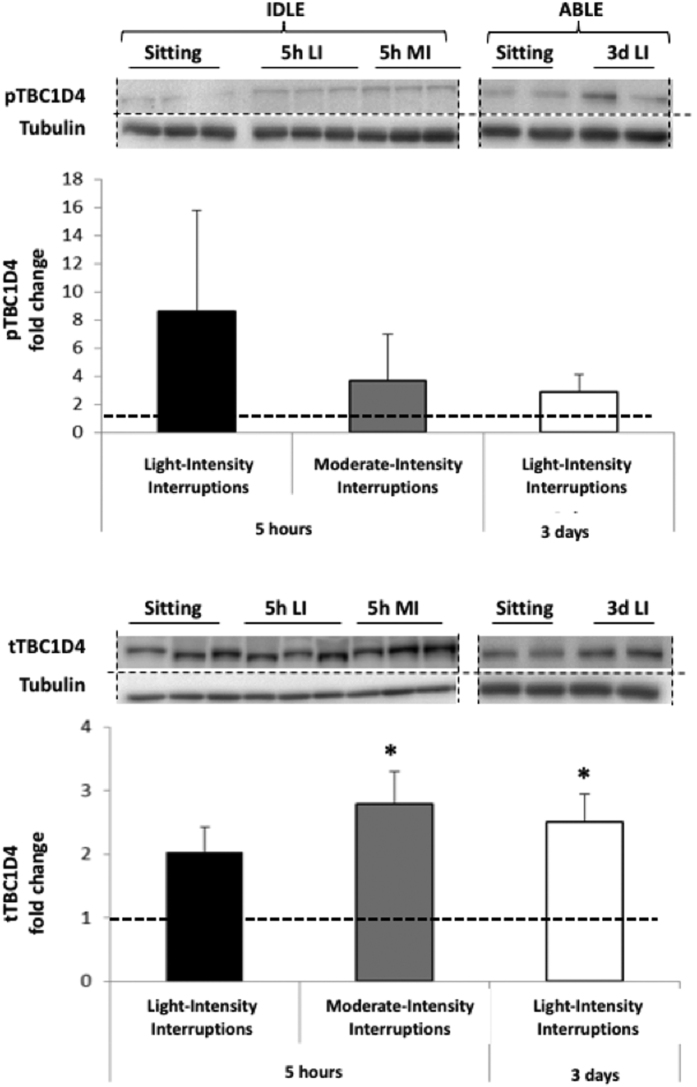
Changes in phosphorylated (Top) and total (Bottom) TBC1D4 protein expression in skeletal muscle following 5 hr light- (5h LI, n = 6) or moderate-intensity (5h MI, n = 6) interruptions, or 3 days of light-intensity interruptions (3d LI, n = 5) compared to uninterrupted sitting (value of 1). Data represent mean ± SEM. Representative blots are shown above the graphs. Dotted lines are showing where the images have been cropped; Full length blots/gels are presented in Supplemental Figure 2 (IDLE, Tubulin), 4 (AIDLE, TBC1D4) and 6 (ABLE, TBC1D4 and Tubulin). Protein expression of TBC1D4 (160 kDa) and tubulin (52 kDa) have been measured on the same blot. All the blots have been run under exact same conditions. *P < 0.05 vs. sitting.

Glucose transport signalling responsiveness was assessed by total and phosphorylated TBC1D4 protein expression (Fig. 5). Despite increases in pTBC1D4 between 2.9 and 8.6 fold, changes did not reach significance for any of the active conditions because of a large inter-individual variability. tTBC1D4 was increased by 5 hr moderate-intensity and 3 days light-intensity interruptions (2.8 ± 1.3, p = 0.018 and 2.5 ± 1.0, p = 0.043, respectively), though this was only a trend for the 5 hr light-intensity interruptions (2.0 ± 1.0, p = 0.2).

### Oxidative phosphorylation pathway (OXPHOS)

**Figure 6:**
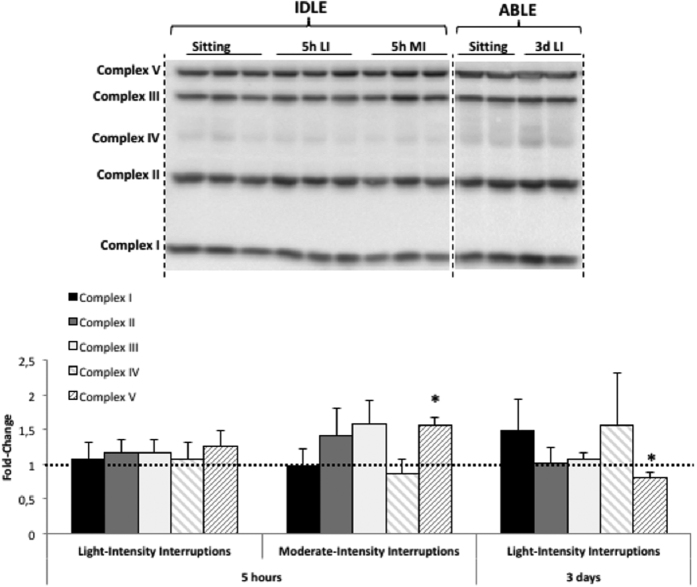
Changes in expression of oxidative phosphorylation (OXPHOS) complexes I to V of the electron transport chain in skeletal muscle following 5 hr light- (5h LI, n = 6) or moderate-intensity (5h MI, n = 6) interruptions, and 3 days of light-intensity interruptions (3d LI, n = 5) compared to uninterrupted sitting (value of 1). Data represent mean ± SEM. Representative blot is shown above the graphs. Dotted lines are showing where the images have been cropped; Full length blots/gels are presented in Supplemental Figure 7 (IDLE and ABLE). Samples from IDLE have been analysed altogether on one blot, samples from ABLE have been analysed altogether on another separate blot. All the blots have been run under exact same conditions. *P < 0.05 vs. sitting.

We investigated changes in protein expression of the mitochondrial oxidative phosphorylation (OXPHOS) complexes in response to acute and short-term interruptions to prolonged sitting. No significant changes were observed in complexes I-IV regardless of the intensity of the interruptions, and the duration of the intervention (Fig. 6). Acute interruptions increased mitochondrial complex V, which may reflect an enhanced capacity for ATP production. Significance was reached with 5 hr of moderate-intensity breaks (+56%, p = 0.043). However, three days of light-intensity interruptions induced a significant reduction in mitochondrial complex V (−20%, p = 0.043).

## Discussion

By examining both the acute and short-term effects of interrupting prolonged sitting with short activity bouts, we showed that 1) both interventions reduce postprandial glucose concentration, 2) acute interruptions to sitting over one day stimulate the contraction-mediated glucose uptake pathway, 3) both acute interruptions to sitting with moderate-intensity activity and light-intensity interruptions over three days induce a transition to modulation of the insulin-signaling pathway, in association with increased capacity for glucose transport. Only the acute moderate-intensity interruptions showed evidence of greater capacity for glycogen synthesis and potentially in ATP production (Table 2).

Glucose uptake into tissues is a tightly regulated process that contributes to glucose homeostasis. Exercise is known to induce many of the same metabolic effects in muscle as insulin, including translocation of the GLUT4 transporter to the membrane, stimulation of glucose uptake, and activation of glycogen synthase^26^,^27^,^28^. In this study we did not observe an up-regulation of the insulin-signaling pathway following one day of interrupting prolonged sitting with bouts of activity, despite a decrease in postprandial plasma glucose concentration. Similarly, it has previously been reported that 30 min of moderate-intensity aerobic exercise in healthy sedentary individuals does not activate the proximal steps in the insulin-signaling cascade, despite whole-body glucose disposal and activated glycogen synthase^29^. In both that study^29^ and our study, measurements were performed about 30–45 min after the last bout of exercise/activity. This suggests that either acute exercise has no effect or that timing of the biopsy was either too early or too late to detect changes in signalling proteins. By contrast, *in vivo* studies have reported that AKT activity is increased in response to skeletal muscle contractile activity in rats when muscle is harvested within 5 minutes^30^,^31^, likely by mechanisms independent of PI3K activation with tyrosine-phosphorylated proteins^32^. Acute AKT activation by muscle contraction in the current study may have been masked by insulin secretion induced by the oral glucose load. In the absence of a control visit with ‘normal’ carbohydrate intake, it is difficult to distinguish between the effect of the 75 g of glucose and of the interventions on total and phosphorylated AKT. Regardless of the composition of the meal, it seems likely that five-six hours after the meal, the influence of nutrient ingestion on the insulin signalling pathway was minimal given that both plasma glucose and insulin concentrations were back to baseline values. In support of this hypothesis, it has been shown in skeletal muscle from rats that the effect of systemic insulin on both AKT activity and phosphorylation lasted 30 minutes only^33^ and in skeletal muscle from humans that after four hours the amount of both insulin receptor substrate 1 (IRS1) and phosphorylated AKT protein was decreased by approximately two compared to the peak observed at 2 hours post meal^34^. In these acute conditions, and based on our observations, glucose uptake seems to be preferentially regulated by the contraction-mediated pathway rather than by the insulin-dependent pathway. Our protocol does not allow us to determine if the increase in contraction signalling was due to the last bout of exercise or to the accumulated effect of the microbouts; further studies will be needed.

Involvement of the contraction pathway with both light and moderate-intensity walking was indicated by significant increases in the downstream target of AMPK, pACC, in association with elevations in tTBC1D4/AS160. This pathway is related to the metabolic status of the muscle cell during contraction and implicates AMPK^35^. AMPK inhibits TBC1D4/AS160 by phosphorylation, which disinhibits GLUT4 translocation to the membrane^20^,^21^, facilitating glucose uptake and its processing to either glycogen synthesis or ATP production. Here we observed a significant increase in both the mitochondrial OXPHOS complex V that may indicate greater ATP production capacity for muscle contraction, and GSK3β that suggests greater capacity for glycogen synthesis with bouts of moderate-intensity activity. We have also previously shown in the same subset of subjects that acute interruption to sitting increases pyruvate dehydrogenase kinase 4 (PDK4) gene expression in a progressive manner between light- and moderate-intensity activity^14^. PDK4 inhibits the pyruvate dehydrogenase complex, a rate-limiting enzyme for glucose oxidation^36^. Nine other genes involved in the regulation of carbohydrate metabolism were also identified in this previous study^14^. Collectively, these findings suggest that acutely interrupting prolonged sitting promotes glucose uptake and favors glycogen synthesis in preference to glucose oxidation. Although activity interruptions of moderate intensity represent a greater metabolic stimulus, light-intensity activity interruptions seem to be sufficient to reduce postprandial glycemia^9^. These changes in glycemia may be the result of muscular contraction *per se* and not from energy demand alone, but further studies will need to confirm this hypothesis.

The well-known changes in glucose metabolism and the oxidative phosphorylation pathway induced by exercise have been associated with an increase in expression of the nuclear factor peroxisome proliferator-activated receptor γ coactivator-1α (PGC1α). One can speculate that frequent interruptions with bouts of activity can similarly activate PGC1α. Two exercise-generated signals can lead to PGC1α activation. The first involves a decrease in ATP and creatine phosphate, which mediates its effect via activation of AMPK. The second exercise-induced signal involves an increase in cytosolic Ca^2+^, which mediates its effect by activating calcium/calmodulin-dependent protein kinase 2 (CAMK2)^37^. Increases in cytosolic Ca^2+^ are triggered by each wave of sarcolemmal depolarization during muscle contraction, and have also been shown to trigger the events that lead to mitochondrial biogenesis and increased GLUT4 expression^38^. This second mechanism may be the primary mechanism at play with light-intensity activity interruptions, while both mechanisms may be activated with moderate-intensity activity interruptions. Greater activation of the intracellular Ca^2+^ release mechanism, due to multiple muscle contractions spread out over the day, may also partially explain why multiple bursts of activity have a greater effect than one single bout of exercise on carbohydrate metabolism. Indeed, regular activity breaks (walking for 1 min 40 s every 30 min) are more effective at decreasing postprandial glycemia and insulinemia in healthy, normal-weight adults than continuous physical activity (walking for 30 min then sitting), compared to 9 hours of sitting^11^. Further investigations are needed to define the respective role of AMPK and Ca^2+^ in the contraction-mediated glucose uptake in response to acute breaking behavior, and this in relationship with both the intensity and type of activity.

After three days of interrupting prolonged sitting by light-intensity activity, there is an activation of the insulin-signaling cascade. These data regarding the activation of the insulin-mediated glucose uptake pathway are in concordance with the metabolic adaptations developed in response to exercise training that result in an increase in insulin-mediated whole-body glucose utilization. After an exercise bout, a decrease in the circulating insulin level is observed and an insulin-sensitive stimulation of glucose uptake in skeletal muscle is induced, which persists 3–6 h after the exercise session^39^. Acute exercise induces glucose transport through GLUT4 translocation, in an additive manner to insulin action in skeletal muscle^40^,^41^. Although significance was not reached, the changes we observed in both total and phosphorylated ACC after three days of light-intensity interruptions suggest that contraction-mediated glucose uptake is not the primary pathway, but is still activated. An increase in pACC may also indicate changes in fat oxidation; further studies will need to assess changes in substrate use in response to interruptions to sitting. Here, we need to acknowledge that the increase in total AKT, GSK3β and TBC1D4/AS160 was not accompanied by a significant increase in phosphorylated AKT, GSK3β and TBC1D4/AS160. Although this discrepancy between the changes in total and phosphorylated proteins is surprising, it is consistent between AKT and two of its downstream targets. Changes in gene transcription, protein translation and post-translational modifications are transient adaptive cellular responses that occur in response to energetic and mechanical challenges imposed by physical activity, which are also transient by nature. The adaptive changes we observed in this study are the results of adaptations in response to repeated very brief bouts of activity and are thus a function of the half-life of the proteins, the transient increase in expression that occurs during recovery from each bout of physical activity and the potential decrease in expression that occurs between two consecutive bouts. By consequence, proteins with longer half-life are more susceptible to accumulate in response to repeated bouts of physical activity than proteins with lower half-life. Some authors reported half-life of phosphorylated and total AKT of 20 and 180 minutes respectively in human colon tumor xenografts^42^. We have not found equivalent data in human muscles but this difference in half-life may partly explain our results. We believe the elevation in total proteins reflects an increase in the capacity for phosphorylation. The dynamic nature of gene transcription and post-transcriptional regulation may also explain the surprising decrease in Complex V protein content observed after 3 days of regular light activity. A differential adaptive response to frequent interruptions of prolonged sitting may also explain this discrepancy between the response to acute and short-term physical activity as it was previously reported for key protein (i.e. PDK4, HKII, GLUT4) and transcriptional regulators (PGC1-alpha, NRF-1, NRF-2, ERR-alpha and Tfam) of skeletal muscle metabolism^43^. Future studies are needed to better define the optimal tissue collection time in order to capture all changes but also to elucidate the potential synergistic effect with diet. Indeed it has been shown that following exercise, re-feeding expended energy and/or carbohydrate reduces or mitigates the effects induced by the exercise on carbohydrate metabolism^44^,^45^.

Interrupting sitting with brief bouts of physical activity confers important health benefits as it mitigates some of the deleterious adaptations to sedentary behavior, i.e. reduced responsiveness of muscle to insulin^46^. This intervention also has great potential for prevention and treatment of T2D. Obese and T2D individuals have reduced whole-body insulin-stimulated glucose disposal^47^, and several lines of evidence suggest that decreased insulin-mediated glucose uptake in muscle is the primary defect in the etiology of T2D^47^,^48^. On the contrary, contraction-mediated glucose uptake has been reported to be preserved in individuals with T2D^49^. Future studies are needed to test these interventions in insulin-resistant individuals.

The controlled study design, including within-subject comparison across three one-day interventions or two three-day interventions administered in random order, the strict supervision of the experimental conditions and the controlled feeding, are strengths of this study. No baseline biopsies were obtained before the oral load to limit the burden on the subjects. By comparing the physically active conditions to the sedentary condition we can however infer the effect of the frequent interruptions of prolonged sitting because the experimental conditions were similar during the different visits with the physical activity regimen the only variable. Another limitation is the small biopsy sample number especially in the study with the two three-day interventions, although it was sufficient to detect significant changes in protein amounts. A larger cohort may have demonstrated larger differences, and given the study greater power to detect statistical significance for the changes where trends were observed. We also acknowledge that measurement of additional parameters would have benefited the present study, but sample volumes obliged us to make strategic choices. The decisions regarding the parameters we measured were based on obtaining the best possible overview of the mechanisms underlying the reduced postprandial glucose concentration observed with acute and short-term interruptions in sedentary time. This study cannot, therefore, provide insight into the chronic effects of interrupting sitting on muscle molecular mechanisms.

## Conclusion

In conclusion, while acute interruptions to sitting stimulate the contraction-mediated glucose uptake pathway, interruptions over three days induce a transition to modulation of the insulin-signaling pathway. These changes suggest a mechanistic basis that may help to explain improved postprandial glucose metabolism with regular interruptions to sitting time. Further mechanistic studies may provide a rational basis for advocacy of breaks from sitting and inform refinement of breaking regimens to maximize their health impact.

## Additional Information

**How to cite this article**: Bergouignan, A. *et al*. Frequent interruptions of sedentary time modulates contraction- and insulin-stimulated glucose uptake pathways in muscle: Ancillary analysis from randomized clinical trials. *Sci. Rep.* **6**, 32044; doi: 10.1038/srep32044 (2016).

## Supplementary Material

Supplementary Information

## Figures and Tables

**Table 1:** Participant characteristics.

Characteristics	IDLE N = 8	ABLE N = 5
Age (years)	55 ± 6	54 ± 6
Weight (kg)	93.8 ± 12.0	89.6 ± 12.0
BMI (kg/m^2^)	31 ± 3	29 ± 2
Fasting insulin (mU/L)	7.0 ± 3.6	9.7 ± 5.1
Fasting glucose (mmol/L)	5.2 ± 0.6	5.3 ± 0.7
HOMA	1.7 ± 1.1	2.3 ± 1.3

Data are presented as mean ± SD. Body Mass Index, BMI.

**Table 2:** Summary of response to acute and short-term interruptions to prolonged sitting compared to uninterrupted sitting condition.

Pathways	Key proteins	5 hr light- intensity interruptions	5 hr moderate-intensity interruptions	3d light- intensity interruptions
Contraction-mediated glucose uptake	pACC	↑	↑	↑ p = 0.08
	tACC	=	=	↑ p = 0.14
Insulin-mediated glucose uptake	pAKT	=	=	↑ p = 0.14
	tAKT	=	=	↑
GLUT4 Translocation	pTBC1D4	=	=	=
	tTBC1D4	=	↑	↑
Glycogen synthesis	pGSK3β	=	=	=
	tGSK3β	=	↑	=
Oxidative phosphorylation	ATPase	=	↑	↓

ACC, Acetyl Carboxylase; ↑, increase; ↓, decrease; =, no statistical change.
