# Supplementary material for: Frequent interruptions of sedentary time modulates contraction- and insulin-stimulated glucose uptake pathways in muscle: Ancillary analysis from randomized clinical trials
Source: Sci Rep. 2016 Aug 24;6:32044. doi: 10.1038/srep32044 (PMC4995429; doi:10.1038/srep32044)
Supplement: Supplementary Information [file srep32044-s1.pdf]

Frequent interruptions of sedentary time modulates contraction- and insulin-stimulated glucose uptake pathways in muscle: Ancillary analysis from randomized clinical trials

Audrey Bergouignan, Celine Latouche, Sarah Heywood, Megan S. Grace, Medini Reddy-Luthmoodoo, Alaina K. Natoli, Neville Owen, David W. Dunstan, Bronwyn A. Kingwell

SUPPLEMENTAL MATERIAL

Supplemental Table 1. Summary of gels shown in the Supplemental Figures.

Gel		pACC	tACC	pAKT	tAKT	pGSK3β	tGSK3β	pTBC1D4	tTBC1D4	Tubulin	OXPHOS
1	Top	IDLE-S1						IDLE-S2			
	Bottom			IDLE-S1		IDLE-S2				IDLE-S3	
2	Top		IDLE-S1						IDLE-S2		
	Bottom				IDLE-S1		IDLE-S2			IDLE-S3	
3	Top	ABLE-S4									
	Bottom				ABLE-S4		ABLE-S5				
4	Top		ABLE-S4								
	Bottom			ABLE-S4		ABLE-S5					
5	Top							ABLE-S6			
	Bottom									ABLE-S6	
6	Top								ABLE-S6		
	Bottom									ABLE-S6	
7											IDLE-S7
8											ABLE-S7

In each case is indicated the study and the supplemental figure number, e.g. IDLE-S1: Samples from IDLE study shown in Supplemental Figure 1.

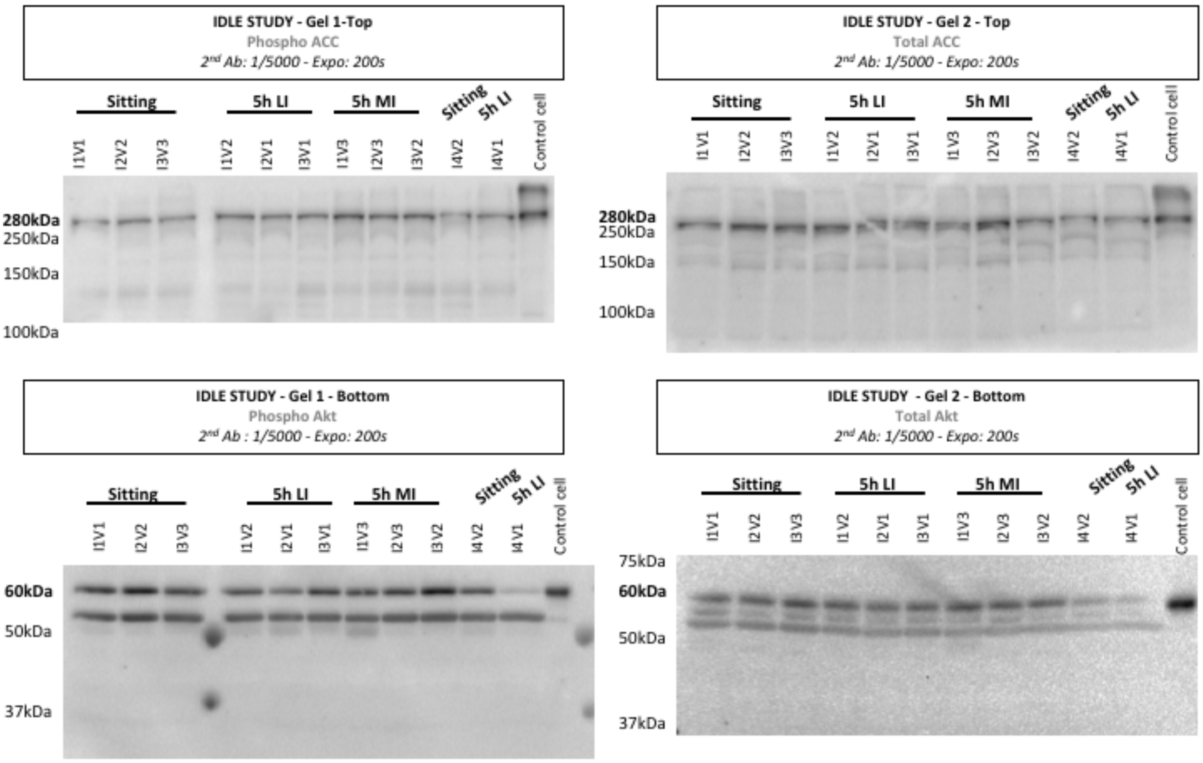

Supplemental Figure 1: Full-length blots/gels showing protein expression of phosphorylated (Gel 1) and total (Gel 2) ACC (Top) and AKT (Bottom) from samples collected in the IDLE study. Gels 1 and 2 were run together. Following migration and transfer, the blots were cut in the middle to allow simultaneous incubation of ACC and AKT anti-bodies. Protein quantitation was corrected for tubulin (See Suppl. Figure 3). The two last samples have not been used in the final analyses because control uninterrupted sitting was missing. This exact figure is the one that has been used in our lab book.

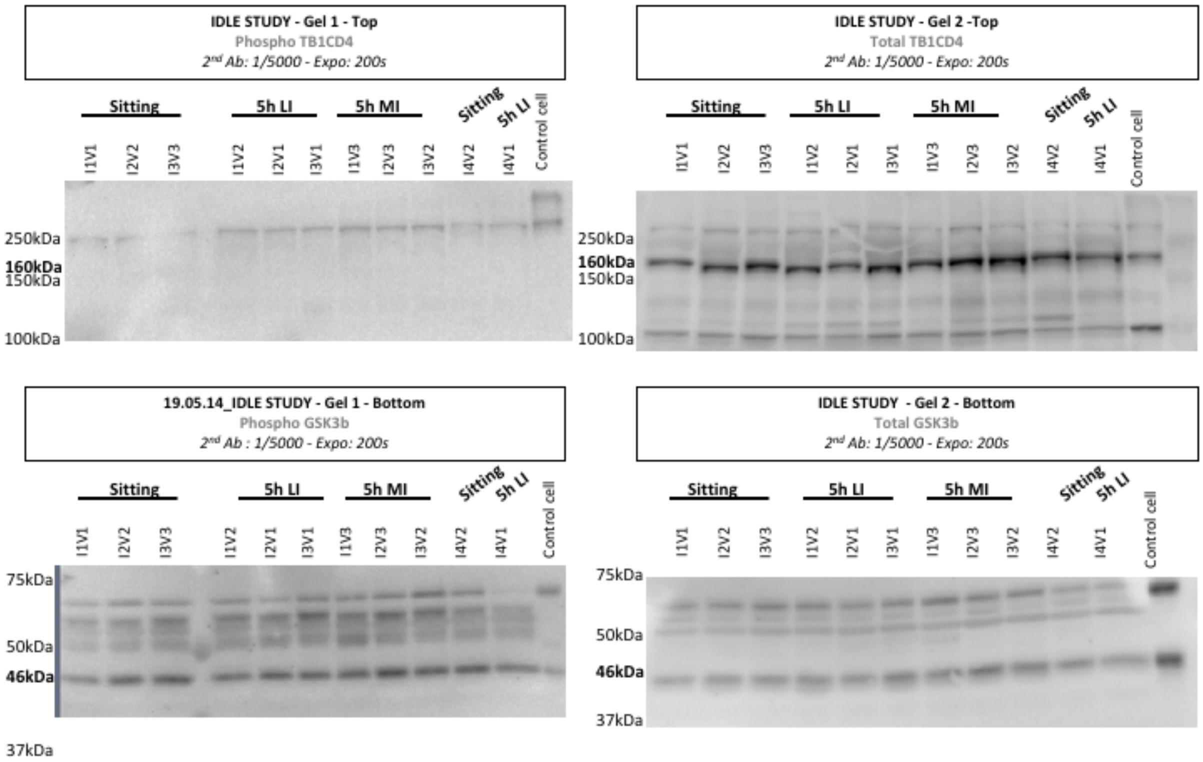

Supplemental Figure 2: Full-length blots/gels showing protein expression of phosphorylated (Gel 1) and total (Gel 2) amount of AS160 (TBC1D4, Top) and GSK3β (Bottom) from samples collected in the IDLE study. Following migration and transfer, the blots were cut in the middle to allow simultaneous incubation of TBC1D4 and GSK3β anti-bodies. Protein quantitation was corrected for tubulin (See Suppl. Figure 3).The two last samples have not been used in the final analyses because control uninterrupted sitting was missing. This exact figure is the one that has been used in our lab book.

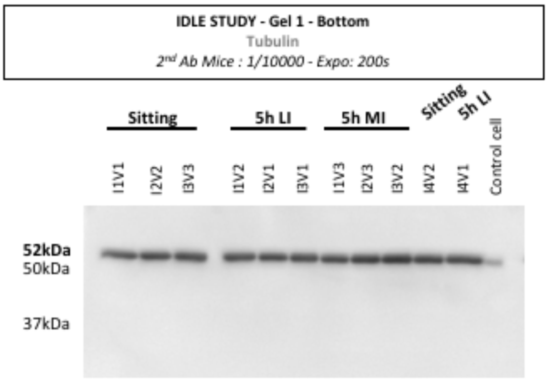

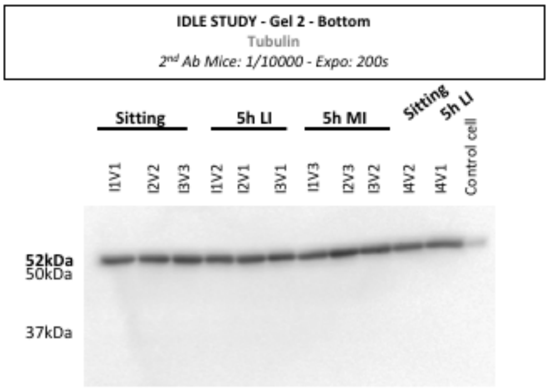

Supplemental Figure 3: Full-length blots/gels showing protein expression of tubulin from samples collected in the IDLE study and loaded on Gels 1 and 2. The two last samples have not been used in the final analyses because control uninterrupted sitting was missing. This exact figure is the one that has been used in our lab book.

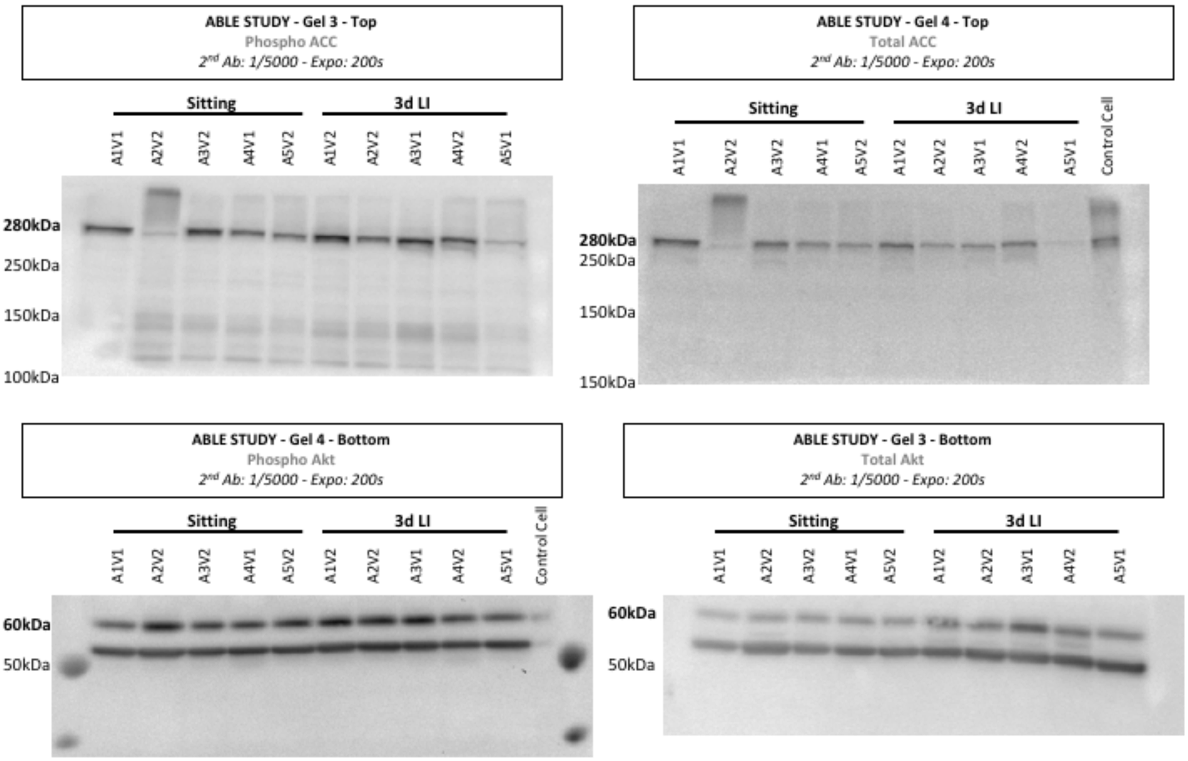

Supplemental Figure 4: Full-length blots/gels showing protein expression of phosphorylated (Gel 3) and total (Gel 4) ACC (Top) and AKT (Bottom) from samples collected in the ABLE study. Following migration and transfer, the blots were cut in the middle to allow simultaneous incubation of ACC and AKT anti-bodies. Protein quantitation was corrected for tubulin (See Suppl. Figure 5, Bottom).This exact figure is the one that has been used in our lab book.

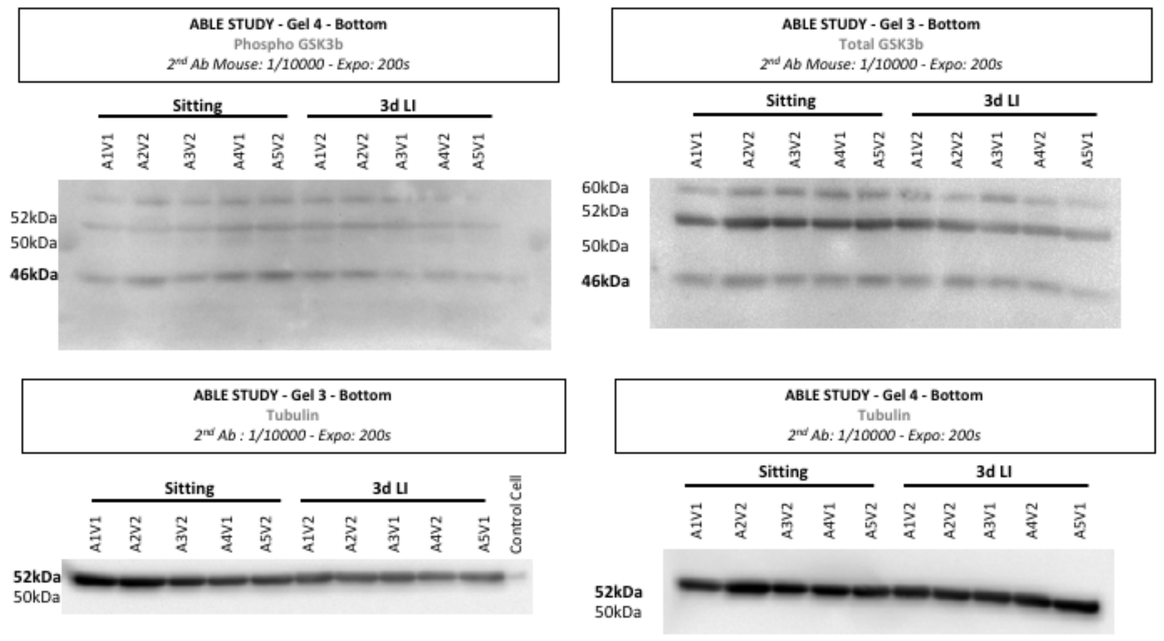

Supplemental Figure 5: Full-length blots/gels showing protein expression of phosphorylated (Gel 4) and total (Gel 3) GSK3β (Top) and tubulin (Bottom) from samples collected in the ABLE study. Protein loading was corrected for tubulin amounts (Bottom). This exact figure is the one that has been used in our lab book.

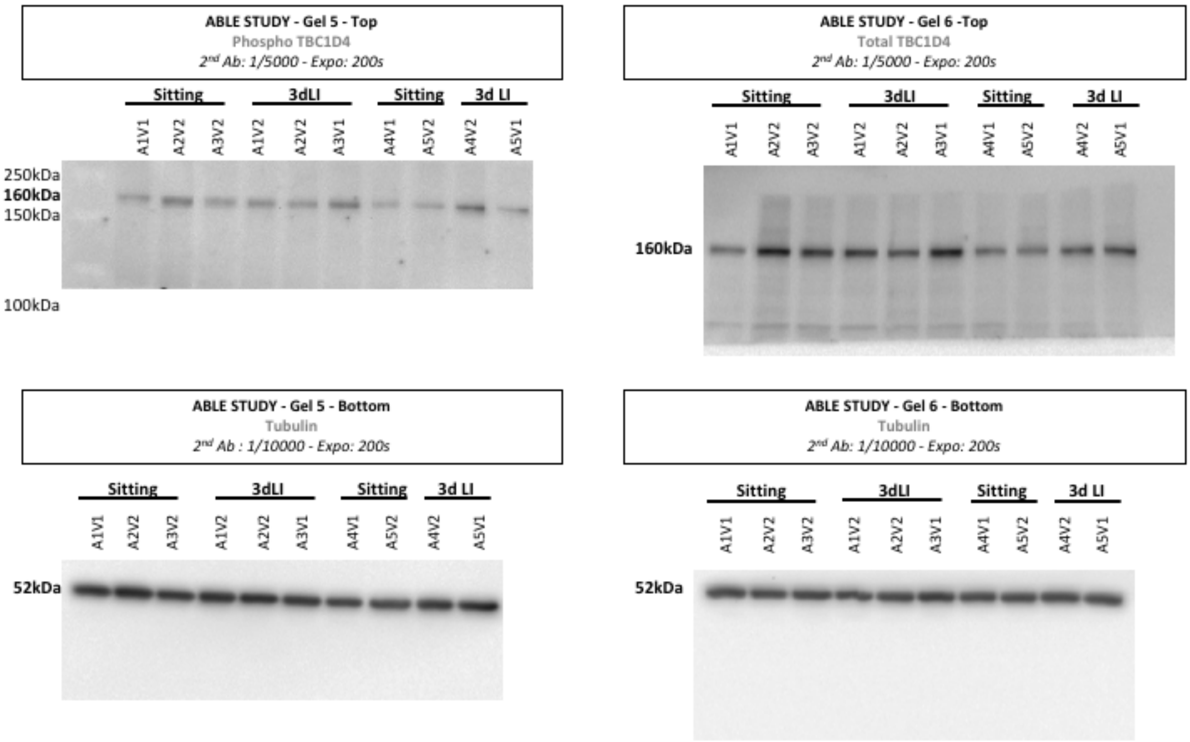

Supplemental Figure 6: Full-length blots/gels showing protein expression of phosphorylated (Gel 5) and total (Gel 6) AS160 (TBC1D4, Top) and tubulin (Bottom) from samples collected in the ABLE study. Following migration and transfer, the blots were cut in the middle to allow simultaneous incubation of TBC1D4 and tubulin anti-bodies. Protein quantitation was corrected for tubulin. This exact figure is the one that has been used in our lab book.

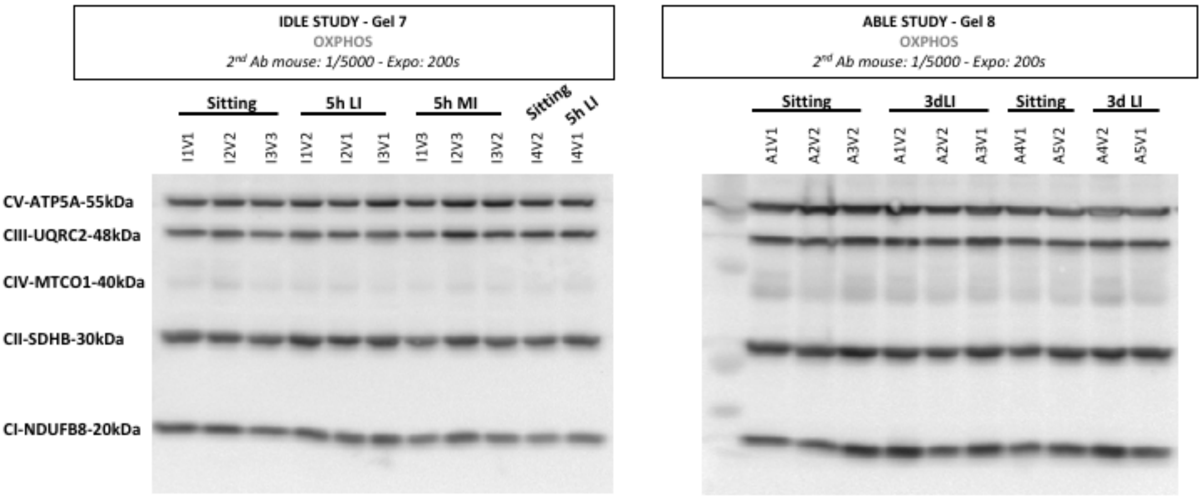

Supplemental Figure 7: Full-length blots/gels showing protein expression of OXPHOS from samples collected in both IDLE (Gel 7) and ABLE (Gel 8) study. Protein loading was not corrected for tubulin amounts. This exact figure is the one that has been used in our lab book.
